# Suppression subtractive hybridization identifies an autotransporter adhesin gene of *E. coli *IMT5155 specifically associated with avian pathogenic *Escherichia coli *(APEC)

**DOI:** 10.1186/1471-2180-10-236

**Published:** 2010-09-09

**Authors:** Jianjun Dai, Shaohui Wang, Doreen Guerlebeck, Claudia Laturnus, Sebastian Guenther, Zhenyu Shi, Chengping Lu, Christa Ewers

**Affiliations:** 1 College of Veterinary Medicine, Nanjing Agricultural University, Nanjing, 210095, China; 2 Institute of Microbiology and Epizootics, Free University Berlin, Berlin, D-10115 Germany

## Abstract

**Background:**

Extraintestinal pathogenic *E. coli *(ExPEC) represent a phylogenetically diverse group of bacteria which are implicated in a large range of infections in humans and animals. Although subgroups of different ExPEC pathotypes, including uropathogenic, newborn meningitis causing, and avian pathogenic *E. coli *(APEC) share a number of virulence features, there still might be factors specifically contributing to the pathogenesis of a certain subset of strains or a distinct pathotype. Thus, we made use of suppression subtractive hybridization and compared APEC strain IMT5155 (O2:K1:H5; sequence type complex 95) with human uropathogenic *E. coli *strain CFT073 (O6:K2:H5; sequence type complex 73) to identify factors which may complete the currently existing model of APEC pathogenicity and further elucidate the position of this avian pathoype within the whole ExPEC group.

**Results:**

Twenty-eight different genomic loci were identified, which are present in IMT5155 but not in CFT073. One of these loci contained a gene encoding a putative autotransporter adhesin. The open reading frame of the gene spans a 3,498 bp region leading to a putative 124-kDa adhesive protein. A specific antibody was raised against this protein and expression of the adhesin was shown under laboratory conditions. Adherence and adherence inhibition assays demonstrated a role for the corresponding protein in adhesion to DF-1 chicken fibroblasts. Sequence analyses revealed that the flanking regions of the chromosomally located gene contained sequences of mobile genetic elements, indicating a probable spread among different strains by horizontal gene transfer. In accordance with this hypothesis, the adhesin was found to be present not only in different phylogenetic groups of extraintestinal pathogenic but also of commensal *E. coli *strains, yielding a significant association with strains of avian origin.

**Conclusions:**

We identified a chromosomally located autotransporter gene in a highly virulent APEC strain which confers increased adherence of a non-fimbriated *E. coli *K-12 strain to a chicken fibroblast cell line. Even though flanked by mobile genetic elements and three different genetic regions upstream of the gene, most probably indicating horizontal gene transfer events, the adhesin gene was significantly linked with strains of avian origin. Due to the nucleotide sequence similarity of 98% to a recently published adhesin-related gene, located on plasmid pAPEC-O1-ColBM, the name *aatA *(APEC autotransporter adhesin A) was adopted from that study.

Our data substantiate that AatA might not only be of relevance in APEC pathogenicity but also in facilitating their reservoir life style in the chicken intestine, which might pave the way for future intestinal preventive strategies.

## Background

*Escherichia coli *typically colonize the mammalian and avian gastrointestinal tract and other mucosal surfaces. While many of these strains are commensal, certain pathogenic strains have the ability to cause severe diseases [1]. Extraintestinal pathogenic *E. coli *(ExPEC) are a group of strains that are implicated in a large range of infections in humans and animals, such as neonatal meningitis, urinary tract infection, intra abdominal infection, pneumonia, osteomyelitis and septicaemia [2-4]. Among the typical extraintestinal infections caused by ExPEC in humans are urinary tract infections (UTIs), which are a major public health concern in developed countries costing healthcare systems billions of dollars annually [5]. Similarly, systemic infection caused by avian ExPEC isolates (avian pathogenic *E. coli*, APEC) is an economically devastating disease to poultry industries [3]. APEC enter and colonize the avian respiratory tract by inhalation of fecal dust leading to localized infections such as airsacculitis and pneumonia. In certain cases, they spread into various internal organs typically causing pericarditis, perihepatitis, peritonitis, salpingitis and other extraintestinal diseases. Systemic infection of poultry is characterized in its acute form by septicemia, commonly resulting in sudden death [3,6].

Previous studies showed that certain subsets of ExPEC strains isolated from different host organisms show high rates of similarity [7-9], envisioning their zoonotic potential, which makes their intensive study even more important.

In general, single ExPEC pathotypes show a high diversity due to differences in the set of virulence genes in their genomes as well as different phylogenetic backgrounds [4]. Thus, unique virulence profiles shared by different human and animal ExPEC pathotypes only rarely exist [7,8].

Although a high number of virulence factors has already been identified, the molecular basis of APEC pathogenesis is not yet fully understood [10,11]. Furthermore, with respect to the unavailability of vaccines eliciting an immune response towards all strains belonging to the highly diverse APEC group, it would be of special importance to identify such virulence factors, which could at the same time, serve as good vaccine candidates. Adhesins, e. g., are known to represent well established targets for the development of vaccines against a number of infectious diseases [12]. Among these bacterial proteins with adhesive properties are autotransporter adhesins, forming a large and diverse family. However, in principal, all members of this family share conserved structural features, that is (i) a secretion signal for the sec pathway in the N-terminus, (ii) a conserved C-terminal translocation domain inserting into the outer membrane of the bacterial cell, and (iii) a variable internal functional passenger domain, which is translocated to the bacterial surface [13]. This process is also known as type V secretion pathway which can involve two proteins, namely a transport and a secreted protein or, as it is the case for autotransporter adhesins, only one protein with dual function [14]. More than 700 members of the autotransporter family are known [15] exhibiting very diverse functions conferred by their surface-exposed passenger domains. They can be involved in proteolysis, cytotoxicity, serum resistance, cell-to-cell spread, autoaggregation, biofilm formation, invasion, and adhesion [13].

Our work focuses on APEC strain IMT5155 and its natural interaction with the chicken host, particularly concentrating on the identification of novel adhesins, conferring the primary and most vital step in the pathogen-host interaction [16]. To further unravel factors possibly involved in APEC pathogenicity, Suppression Subtractive Hybridization was applied to the genome of APEC strain IMT5155 and human UPEC strain CFT073, both allocated to different multilocus sequence type complexes, leading to the identification of the putative APEC autotransporter adhesin gene *aatA*. A 98% identical gene was also found in strain APEC_O1 by Li and co-authors only recently [17]. The respective gene *aatA *and its localization in the IMT5155 genome was analyzed and compared with similar loci present in sequenced *E. coli *genomes. To verify the functional role of the putative adhesin *in vitro *adhesion assays were performed using DF-1 chicken fibroblast cells. In addition, a representative collection of ExPEC and commensal *E. coli *strains from different hosts and phylogenetic groups was screened for the presence of the adhesin gene to determine whether it is associated with specific pathotypes or phylogenetic groups.

## Results

### Identification of genes present in APEC strain IMT5155 but absent in human UPEC strain CFT073

The aim of the work presented here was to identify new potential virulence genes specific for avian pathogenic *E. coli *(APEC) strains, which might be important in the pathogenesis of systemic infections in poultry and helpful in delineating this pathotype from other ExPEC pathotypes. By applying Suppression Subtractive Hybridization (SSH) to APEC strain IMT5155 and human ExPEC strain CFT073, 96 clones were obtained from the not yet sequenced APEC strain IMT5155 which were not present in the archetypical UPEC strain CFT073. These 96 clones were amplified by PCR and cloned into plasmid pCR2.1. To explore the specificity of these gene fragments for APEC strain IMT5155, PCR amplicons were transferred to a nylon membrane and southern hybridization analysis was performed with labelled genomic DNA of UPEC strain CFT073 and K-12 strain MG1655, respectively. Among the 96 clones, 34 contained an insert neither hybridizing with the labelled genomic DNA of CFT073 nor with that of K-12 strain MG1655. Thus, a total of 34 DNA fragments supposed to be specific for IMT5155 were sequenced [GenBank: AM230450 to AM230483]. Subsequent BLAST analyses revealed that 28 DNA fragments, ranging from 100 bp to 1000 bp in size, were indeed absent from the genome of CFT073 and K-12 strain MG1655 and were regarded as specific for APEC strain IMT5155 in the experimental approach. Sequences of the identified loci and their corresponding products, respectively, show similarities to bacteriophages; EntS/YbdA MFS transporter proteins, conjugal transfer proteins; restriction modification enzymes and different biosynthesis enzymes, e.g. a polysialic acid biosynthesis protein, a poly-alpha-2,8 sialosyl sialyltransferase, a phosphoglycerate dehydrogenase, a dTDP-rhamnosyl transferase and a glycosyltransferase. Nine of the identified fragments were similar to sequences encoding proteins of unknown function.

One of the SSH fragments (namely B11, with a size of 225 bp, GeneBank AM230456.1) was of special interest to us, as its potential product showed sequence similarity to proteins of the family of bacterial autotransporter adhesins, including a putative adhesin gene located on pAPEC-O1-ColBM plasmid, which at the time of our analysis, was not described in detail. Our further analyses focused on this gene. A 6,154 bp sequence of IMT5155 containing the open reading frame and the flanking regions of the gene was submitted to GenBank [GU550065]. According to the nucleotide sequence similarity of 98% to the previously described adhesin gene *aatA *(APEC autotransporter adhesin A), which is located on plasmid pAPEC-O1-ColBM [18], we adopted the name and focussed our further study on a detailed characterization of IMT5155 AatA.

### Sequence analysis of the autotransporter adhesin gene *aatA*

**Figure 1:**
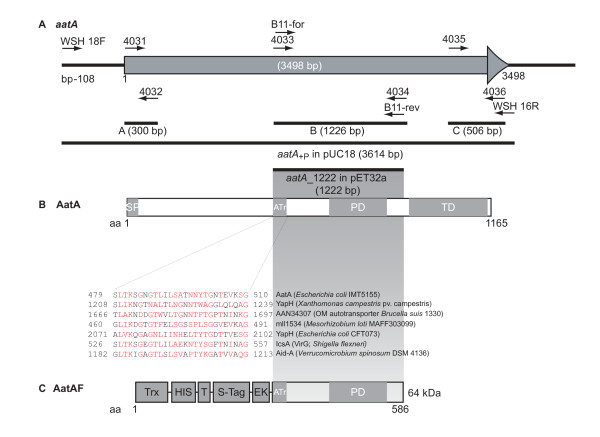
**APEC IMT5155 *aatA*: genomic locus and predicted protein structure**. **A: Scheme of the genomic locus of *aatA *in IMT5155**. The open reading frame (ORF) of *aatA *is indicated as grey arrow. Black bars indicate PCR fragments amplified using oligonucleotides marked as black arrows. The size of each fragment and the ORF are given in brackets. **B: Scheme of the predicted protein AatA**. The 3,498 bp ORF results in the 124-kDa APEC autotransporter adhesin A. Sequence analyses revealed the given domain structure. At the N-terminus a signal peptide (SP) is predicted which probably enables the sec machinery to secrete AatA across the cytoplasmic membrane. The autotransporter repeat (ATr) is found in many AT adhesins and proteins, which are predicted as AT adhesins. The alignment below the protein structure shows conserved amino acid (aa) residues within one AT repeat. C-terminal of the AT repeat lies the predicted functional passenger domain found in AT adhesins (PD). The AT-adhesin-typical translocation domain (TD) resides at the C-terminus of the protein. **C: Scheme of fusion protein AatAF**. Using oligonucleotides B11-for and B11-rev the 1,222 bp fragment aatA_1222, comprising the region for the AT repeat and the functional PD was amplified by PCR and cloned into pET32a(+) for expression. The 64-kDa fusion protein AatAF contains an enterokinase recognition site (EK), an S tag, a thrombin site (T), a His_6 _tag and a thioredoxin tag (Trx) fused to the N-terminus of the adhesin peptide to enhance protein solubility and to simplify protein purification.

**Figure 2:**
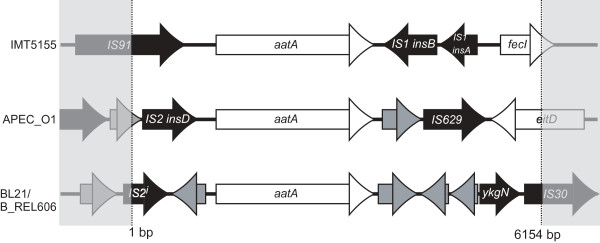
**Comparison of the genome regions surrounding *aatA *of IMT5155, APEC_O1, B_REL606 and BL21**. In total we sequenced 6,154 bp of the strain IMT5155 including the *aatA *gene, 1,072 bp upstream and 1,584 bp downstream of *aatA*. Our sequence was compared with the comparable 6,154 bp genome regions of the sequenced strains harbouring *aatA *homologs: APEC_O1, B_REL606 and BL21. Open reading frames (ORFs) are shown as arrows. White arrows represent known genes, predicted ORFs are shown in grey and insertion sequences or an ORF encoding a putative transposase are indicated in black. IS2^i^: interrupted insertion sequence.

To determine the complete sequence of *aatA *and its flanking region we generated a cosmid library of APEC strain IMT5155. This library was screened by PCR using three different oligonucleotide pairs (4031 to 4036, see Additional file 1: Table S1). After identification of the *E. coli *clone containing a cosmid with the *aatA *sequence, the cosmid DNA was isolated and sequenced. Double strand sequence information was obtained for the complete predicted open reading frame (ORF; Figure 1A) of *aatA *(3,498 bp) and 2,656 additional nucleotides of the surrounding region. MegaBlastN analyses revealed a 98% sequence identity of this ORF with a coding sequence from *E. coli *APEC_O1 (Acc. No. NC_009837.1; locus pAPEC-O1-ColBM [18]). In addition, homologues were also found in *E. coli *strain BL21(DE3) (NC_012947.1; locus ECBD_0123) and *E. coli *strain B_REL606 (NC_012967.1; locus ECB_03531) showing a 99% identity to *aatA*. The coverage for the 98 to 99% identical region was 100% in BL21, B_REL606, and APEC_O1, respectively. Figure 1A gives an overview of the genomic locus of IMT5155 containing the *aatA *ORF. Figure 2 shows the comparison of the 6,154 bp genome regions of the strains containing *aatA*. The schematic view of the genome loci reflects similarities and differences among the sequenced *E. coli *strains harbouring *aatA*. As illustrated in this figure, the ORF of the adhesin gene is conserved among IMT5155, APEC_O1, BL21, and B_REL606, whereas the surrounding regions differ, except for BL21 and B_REL606 which show 100% identity in this region. Further analysis of the sequences up- and downstream of *aatA *showed that in the strains mentioned above the 5' as well as the 3' flanking regions encode mobile elements (Figure 2). Among these are sequences similar to insertion sequence IS2 and IS91 in the 5' flanking region of *aatA *and genes coding for insertion sequences IS1, IS30 and IS629 in the 3' flanking region, respectively. The presence of genes encoding transposases in all four strains suggests that *aatA *has been acquired by horizontal gene transfer.

Sequence analyses also revealed that *aatA *is likely to be a single gene locus and not part of an operon. This is in accordance with data of other autotransporter adhesins [13,19]. Promoter prediction analysis with 200 bp upstream of the ATG showed two possible transcriptional start sites at position -59 (p = 0.97) and -86 (p = 0.97) relative to the ATG of the *aatA *ORF in IMT5155. This 200 bp region is almost identical in APEC_O1 (except one bp exchange and one nucleotide deletion). The most likely transcriptional start site is predicted at position -85 (p = 0.97) relative to the ATG of the *aatA *ORF. The 200 bp region upstream of *aatA *in strains BL21 and B_REL606 shows only 70% identity to the respective region in APEC strain IMT5155. A possible transcriptional start site was predicted at position -54 (p = 1.0) relative to the ATG. Expression analyses in later studies showed that the promoter of the IMT5155 *aatA *lies within the 100 bp upstream of the ORF (see below).

### *aatA *is plasmid-encoded in APEC_O1 but not in APEC strain IMT5155

Although APEC strains APEC_O1 and IMT5155, both assigned to multi locus sequence type complex (STC) 95, are closely related the surrounding regions of *aatA *significantly differ in these strains. The genome sequence of APEC_O1 reveals that the *aatA *homolog in this strain is located on the 174,241 bp pAPEC-O1-ColBM plasmid, downstream of the *eitABCD *operon [18]. Sequence analysis of the IMT5155 ColV plasmid p1ColV_5155 _(about 181 kb) as well as of the second IMT5155 plasmid p2_5155 _(4.6 kb) (U. Böhnke and C. Ewers, unpublished data) showed that *aatA *is not plasmid-located in IMT5155.

### *aatA *encodes a protein with features of an autotransporter

BLASTX analyses with the IMT5155 *aatA *ORF revealed that the potential AatA protein comprises a signal peptide at the N-terminus as predicted with the SignalP 3.0 Server; an autotransporter repeat conserved among autotransporter adhesins from different bacterial species; a passenger domain and a C-terminal translocation domain (Figure 1B and Table 1). According to these data, *aatA *likely encodes an adhesin of the autotransporter family.

**Table 1:** BlastX analyses using the *aatA *sequence (3,498 bp) of *Escherichia coli *strain IMT5155

Accession number	Similar protein	Microorganism	Similarity
ZP_03068020.1	Putative autotransporter adhesin	*E. coli *B_REL606	99%
YP_003034319.1	Predicted outer membrane autotransporter barrel domain protein	*E. coli *BL21(DE3)	99%
YP_001481251.1	Putative autotransporter adhesin	*E. coli *APEC_O1	98%
NP_061407.1	Putative autotransporter adhesin	Plasmid F *E. coli *K-12 strain	47%
YP_001452019.1	Putative autotransporter adhesin	*Citrobacter koseri* ATCC BAA-895	42%
NP_286049.1	Putative beta-barrel outer membrane protein	*E. coli *O157:H7 EDL933	42%
NP_308389.1	AidA-I adhesin-like protein	*E. coli *O157:H7 str. Sakai	42%

**Figure 3:**
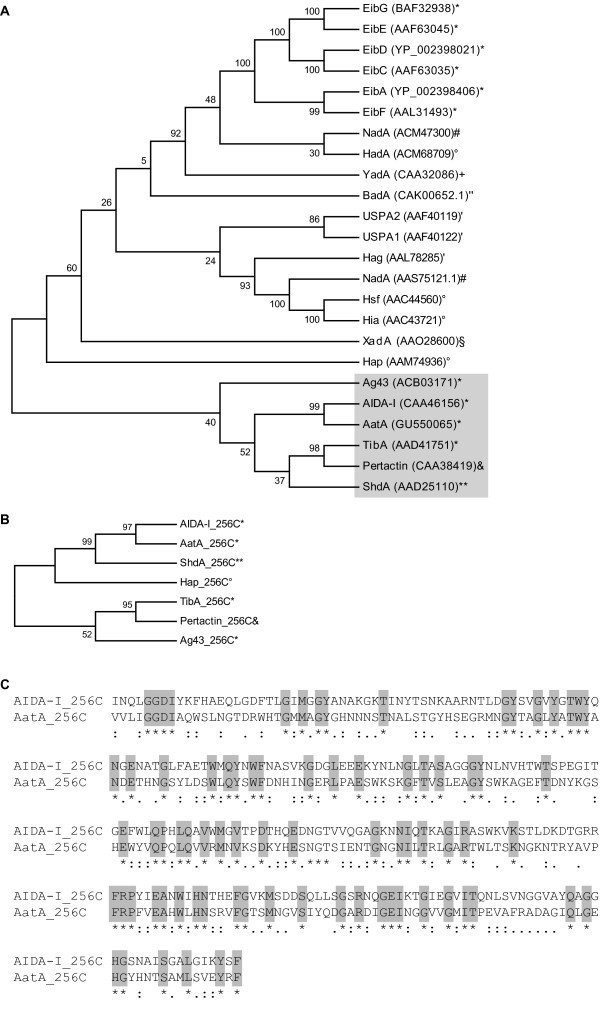
**Phylogenetic tree of autotransporter adhesins including AatA**. The phylogenetic trees were calculated with the Neighbor-Joining-Algorithm on the basis of a ClustalW multiple alignment of 24 protein sequences from known adhesins of the autotransporter family including AatA. The percentages of replicate trees in which the associated taxa clustered together in the bootstrap test (1000 replicates) are shown next to the branches. Protein sequences were obtained from the NCBI database. **A: **Phylogenetic tree (NJ-tree) obtained using the complete 24 protein sequences. **B: **NJ-tree obtained using only the last 256 amino acid residues according to the smallest protein HadA in ClustalW analyses. Here, only proteins clustering in one phylogenetic branch with AatA are shown. **C: **The amino acid residue alignment of the C-termini of AIDA-I and AatA are shown highlighting identical residues (*indicates fully conserved residues, :indicates fully conserved strong groups, .indicates fully conserved weaker groups). Symbols indicate the species: *Escherichia coli, ^#^*Neisseria meningitidis*, °*Haemophilus influenzae*, ^+^*Yersinia enterocolitica*, '*Moraxella catarrhalis*, ´´*Helicobacter pylori*, ^$^*Xylella fastidiosa*, **Salmonella Typhimurium, and ^&^*Bordetella pertussis*.

Thus, the relation of this protein to other autotransporter family members was further investigated. ClustalW http://align.genome.jp/ analyses were performed with 24 protein sequences from already known adhesins of the autotransporter family including proteins from *E. coli*, *Neisseria meningitidis*, *Haemophilus influenzae*, *Yersinia enterocolitica*, *Moraxella catarrhalis*, *Helicobacter pylori*, *Xylella fastidiosa*, *Salmonella *Typhimurium, *Bordetella pertussis *and the newly identified *E. coli *IMT5155 adhesin AatA (Figure 3). Protein sequences were obtained from the NCBI database and the respective Accession numbers are given in Figure 3. The results presented as phylogenetic tree (N-J tree) show that AatA clusters within one group together with AIDA-I (adhesin involved in diffuse adherence), TibA (toxigenic invasion locus B protein A) and Ag43 (antigen 43) from *E. coli*, which are closely related to ShdA (similar to the C-terminal region of AIDA; IcsA) from *Salmonella *and Pertactin from *Bordetella*. We also investigated the 24 adhesins including only their last 256 amino acid residues according to the smallest protein HadA to circumvent false results due to different protein lengths. The results were comparable to those of the analyses of the complete protein sequences. Similarly, comparing only the C-termini, AIDA-I clusters in one phylogenetic branch with AatA, thus the C-terminus of AatA seems to be most related to that of AIDA-I (Figure 3B). The amino acid residue alignment of the C-termini of AIDA-I and AatA revealed a number of identical residues as shown in Figure 3C. Comparing only the C-terminus one has to keep in mind that this part contains the transmembrane domain to span the bacterial membrane, thus it is likely to be the most conserved part among all autotransporter adhesins.

**Figure 4:**
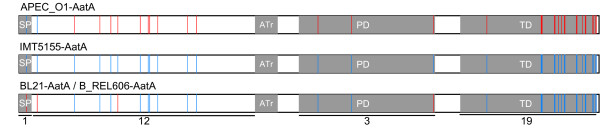
**Comparison of the AatA proteins of IMT5155, APEC_O1, BL21, and B_REL606**. AatA amino acid sequences were compared using MegAlign (Lasergene 6, DNASTAR, WI, USA). Proteins are depicted as schemes indicating specific protein domains as predicted (SP: signal peptide; ATr: autotransporter repeat region; PD: passenger domain; TD: transmembrane domain). Amino acid differences are shown as lines. Red lines indicate differences to the IMT5155-AatA amino acid sequence. The total number of amino acid substitutions is given for each protein domain below the protein schemes.

We also examined the amino acid differences of the conserved AatA proteins in *E. coli *IMT5155, APEC_O1 and BL21 and B_REL606, respectively. The AatA of the latter two strains are 100% identical. In total, 19 amino acid substitutions were found in the C-terminus containing the transmembrane domain; 3 variable positions lie within the passenger domain and 13 differences in amino acid sequence were found in the N-termini of the AatA proteins (Figure 4). Interestingly, the transmembrane domains of BL21 and IMT5155 are 100% identical and the 19 C-terminal amino acid differences occur in APEC_O1 compared to these two strains. Also the majority of amino acid substitutions within the N-terminus (10 of 13) occur in APEC_O1 in contrast to the almost identical AatA proteins from BL21 and IMT5155 (only 3 substitutions). Taken together, the adhesins of the two APEC strains differ more than the AatA proteins of IMT5155 and the non-pathogenic BL21 strain. It is notable that most differences are present in the C-terminus which forms a pore through the membrane and is thus functionally conserved whereas the passenger domains are known to be variable among the autotransporter adhesin family members.

### *aatA *is expressed in APEC IMT5155

To determine whether *aatA *is transcribed in wild-type strain IMT5155 under laboratory conditions, its expression was studied by quantitative real-time PCR including *aatA*-negative UPEC strain CFT073 and *aatA*-positive strains BL21 and APEC_O1. Expression of *aatA *was detectable in all *aatA*-positive strains after growth in LB. Interestingly, our analysis revealed different transcriptional levels of *aatA *in IMT5155, APEC_O1 and BL21 when compared to the constitutively expressed housekeeping gene *gyrB*. In detail, we observed an increased transcription of *aatA *in APEC_O1 (2.71 ± 0.33 fold change), while BL21 showed a considerable lower transcription level of this gene (0.16 ± 0.33 fold change) as compared with the transcription level determined for *aatA *in IMT5155. As expected no specific transcription was detected for *aatA *in CFT073 (fold change < 0.0001).

### AatA triggers antibody production in rabbits

To investigate if the *aatA *transcript in IMT5155 is indeed translated into the expected AatA protein, a specific antibody against AatA was raised.

**Figure 5:**
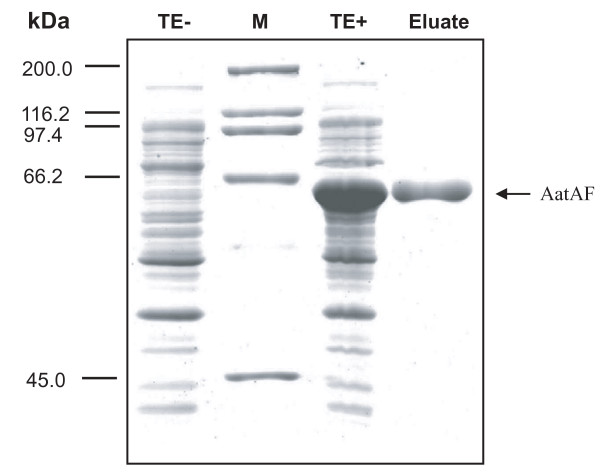
**Purification of AatAF after expression in *E. coli *BL21**. The internal part of *aatA *encoding the passenger domain of AatA was cloned into pET32a(+) leading to the expression of the 64-kDa fusion protein AatAF. BL21 cells were incubated in LB at 37°C without (lane 1) or with (lane 3) addition of IPTG. Proteins of total extracts (lane 1 and 3) and of eluates of the purified AatAF (lane 4) were separated on an SDS-PAGE and stained with coomassie. TE: total extract, -: without IPTG, +: with IPTG; M: protein marker.

For the production of specific AatA antibodies we cloned the internal part of *aatA *(1,222 bp from position 1,375 bp to 2,596 bp within the ORF) into expression vector pET32a(+) under the control of the IPTG-inducible T7 promoter (see Figure 1). The resulting construct led to the expression of a 64-kDa fusion protein in *E. coli *BL21 designated AatAF (see Figure 1C for overview). Figure 5 shows a coomassie stained SDS-PAGE, demonstrating that AatAF was well expressed in *E. coli *BL21 after induction with IPTG (compare lane 1 and 2) and successfully purified using the HisTrap column (lane 3). The purified protein was then used to produce specific AatA antibodies as described in methods.

**Figure 6:**
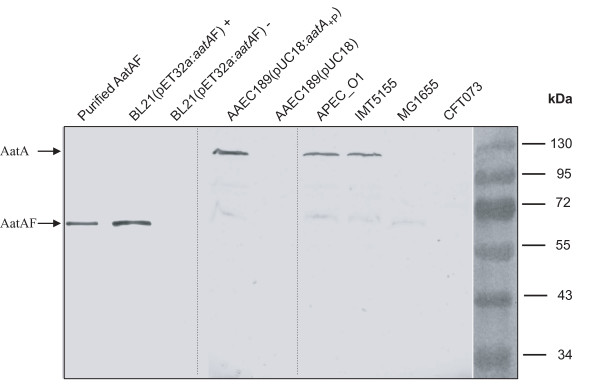
**Expression of AatA in different *E. coli *strains**. The purified fusion protein (lane 1) and total protein extract of BL21(pET32a:*aatA*F) (lanes 2 and 3), expressing AatAF under the control of the IPTG-inducible promoter, AAEC189(pUC18:*aatA*_+P_) expressing *aatA *under the control of the native promoter and AAEC189(pUC18) (lanes 4 and 5), APEC_O1 (lane 6), IMT5155 (lane7), CFT073 (lane 8) and MG1655 (lane 9) were separated on an SDS gel and blotted to polyvinylidene fluoride membrane. The membrane was then incubated with anti-AatA antibody.

To confirm that the produced antibody is specific and able to recognize not only the fusion protein AatAF but also the native wild-type protein AatA, total protein extract of the strain BL21(pET32a:*aatA*F) prior and after induction of the IPTG-inducible promoter as well as the purified fusion protein AatAF and total protein extracts of strains IMT5155, APEC_O1, CFT073 and MG1655 were separated on an SDS gel and transferred to a polyvinylidene fluoride membrane. As shown in Figure 6 incubation with anti-AatA indeed led to the detection of protein bands of the expected size for AatAF in the total extract of BL21(pET32a:*aatA*F) and wild-type AatA protein in APEC strains IMT5155 and APEC_O1, respectively. As expected, no signal was observed for CFT073 and MG1655, which have no *aatA *homolog in their genomes. Taken together our data show that AatA is suitable for the production of specific antibodies. Furthermore, this antibody recognizes wild-type AatA protein, demonstrating that APEC strains IMT5155 and APEC_O1 express a protein of the expected size, thus the gene in their genomes is likely to encode a functional adhesin. Surprisingly, no band of the expected size for AatA was detectable in strain BL21, which might be due to several reasons, including the lower transcription of the gene in this strain probably due to the presence of the different promoter region as compared to the APEC_O1 and IMT5155 *aatA *promoter regions.

### Expression of AatA in the *fim *negative *E. coli *strain AAEC189 leads to enhanced adhesion abilities

**Figure 7:**
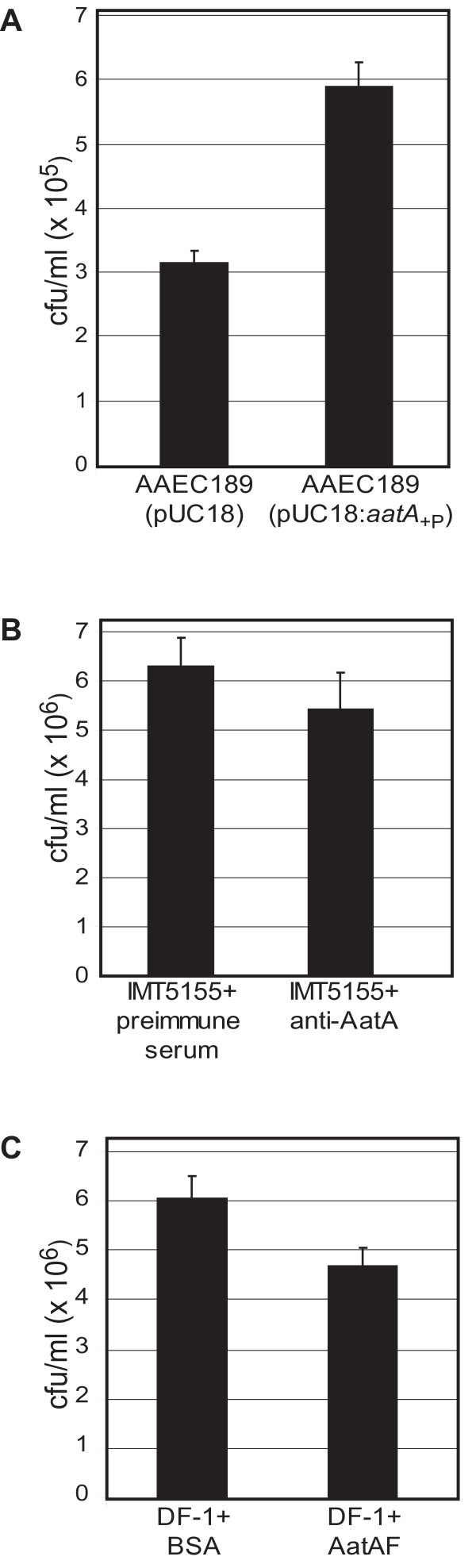
**AatA plays a role in adhesion to chicken fibroblast DF-1 cells**. **A: Adhesion of AAEC189(pUC18) and AAEC189(pUC18:*aatA*_+P_), expressing *aatA *under its native promoter, to DF-1 cells**. Monolayers of DF-1 cells were incubated with *E. coli *strains for 3 h at 37°C. Adherent bacterial cells were harvested and the number was determined. **B: The anti-AatA antibody inhibits binding capacity of IMT5155 to DF-1 cells**. IMT5155 was incubated with preimmune serum (control) and with anti-AatA antibody, respectively. After washing, bacteria of each experiment were added to monolayers of DF-1 cells and incubated for 3 hours. Adherent bacterial cells were harvested and the number was determined. **C: Pre-incubation of DF-1 cells with AatAF protein reduces adhesion capacity of IMT5155 to these cells**. Confluent monolayers of DF-1 cells were incubated with BSA (control, 50 μg/well) or purified and refolded AatAF protein (50 μg/well) for 1 h at 37°C prior to the addition of IMT5155 cells. After 3 h of incubation, adherent bacterial cells were harvested and the number was determined. **A-C: **Columns represent the mean value of three independent wells per strain. Standard errors of the mean values are indicated as error bars. The experiment was repeated three times showing comparable results.

Based on sequence analyses it was assumed that also the chromosomal *aatA *variant encodes a protein with adhesive function. To verify this, adhesion assays were performed using the chicken embryo fibroblast cell line DF-1. For this, *aatA *was expressed under control of its native promoter in *E. coli *strain AAEC189. AAEC189 is an MG1655 strain in which the *fim *operon is deleted leading to a reduced adhesion in *in vitro *assays [20]. AAEC189(pUC18:*aatA*_+P_) and the control strain AAEC189(pUC18) were incubated with DF-1 cells for 3 h. As shown in Figure 7A, the *aatA *containing strain displayed a 1.9 fold increase in adherence as compared to the adhesion of the negative control (*P *= 0.009). This suggests that AatA mediates adhesion of *E. coli *cells to chicken cells. To prove that AatA is indeed expressed, total extracts of AAEC189(pUC18) and AAEC189(pUC18:*aatA*_+P_) were analyzed by immunoblot with anti-AatA antibody. As shown in Figure 6 (lane 4) a specific protein band appears for strain AAEC189(pUC18:*aatA*_+P_), which has the expected size of AatA. Taken together, our data demonstrate that AAEC189(pUC18:*aatA*_+P_) expresses AatA wild-type protein, which leads to enhanced adhesion of AAEC189. Thus, we can assume a role of AatA in adhesion of *E. coli *to chicken cells. Furthermore, our data show that the *aatA *promoter lies within the 100 bp upstream of the gene.

### AatA is involved in adhesion of APEC strain IMT5155

In order to investigate the role of AatA in adhesion of the wild-type APEC strain IMT5155 we have chosen an adhesion inhibition approach using specific anti-AatA antibody for pre-treatment of bacteria prior to incubation with DF-1 cells. Bacteria pre-treated with pre-immune serum served as control. As shown in Figure 7B the anti-AatA antibody slightly reduced *E. coli *IMT5155 adherence to DF-1 cells, which indicates that AatA might play a role in adhesion of IMT5155 to eukaryotic cells. However, the difference is rather low. This observation is probably due to the number of other adhesins present in IMT5155, which are not blocked by the anti-AatA antibody and thus are still able to mediate adhesion.

In a second complementary adhesion inhibition approach DF-1 cells were pre-incubated with purified and refolded AatAF protein and with BSA as negative control, respectively, prior to the adhesion assay. Bacterial cells, which adhered to the pre-treated DF-1 cells, were harvested and the number of adherent bacteria was determined. As shown in Figure 7C, the incubation of DF-1 cells with AatAF led to a reduction in adhesion of IMT5155 as compared to cells pre-incubated with BSA. As described above, IMT5155 expresses AatA under the growth conditions used for adhesion assays. In conclusion, our results indicate that AatA plays a role in adhesion of IMT5155 to chicken cells.

### Distribution of *aatA *among 779 ExPEC isolates with regard to pathotype, host, and ECOR group

Out of a total of 779 *E. coli *tested, 186 isolates (23.9%) were found to be positive for *aatA *(Table 2). Turning our attention to APEC strains, we found that 32.7% of 336 isolates harboured *aatA *(*P *< 0.001), while the gene was less frequently observed among UPEC (4.7%) and other ExPEC (9.1%) isolates and completely absent in NMEC strains. Interestingly, a high percentage (28.9%) of commensal strains, in particular of avian sources (56.3%; *P *< 0.001) was positive for *aatA*. Taking a closer look at the association of the host and the presence of *aatA *in ExPEC strains, we observed that 38.4% (n = 168) of avian strains harboured the gene, accounting for 90.3% of all 186 *aatA *positive strains. Essentially minor percentages of *aatA*-positive strains were recovered from companion animals (3.2%) and humans (5.1%), while among various non-avian hosts, only pigs and cattle also infrequently possessed *aatA *(other animals: 16.7%). Statistical analyses confirmed a positive correlation of *aatA*-possessing strains to birds and a negative correlation to strains from humans and companion animals (both *P *< 0.0001).

**Table 2:** Distribution of *aatA *among 779 extraintestinal pathogenic and commensal *Escherichia coli *strains

	Total no. of strains per group	Strains positive for *aatA*	
		
		**No**.	%
**All strains**	779	186	23.9

**Pathotype/*E. coli *group**			

APEC	336	110	32.7
UPEC	149	7	4.7
NMEC	25	0	0
other ExPEC	44	4	9.1
Commensals	225	65	28.9
Bird	103	58	56.3
Non-avian animals	33	4	12.1
Human	89	3	3.4

**Host**			

Bird	438	168	38.4
Human	212	9	3.2
Companion animals	93	3	3.2
Other animals	36	6	16.7

**ECOR group**			

A	217	49	22.6
B1	115	31	27.0
B2	314	54	17.2
D	133	52	39.1

Although *aatA *was detected in strains of all major phylogenetic groups, the highest percentage of positive strains was observed in ECOR group D (39.1%; *P *< 0.001) and in descending order in groups B1 (27.0%), A (22.6), and B2 (17.2%) (Table 2). The frequent presence of *aatA*-positive strains within ECOR group D is even more remarkable if we merely consider avian strains, whether pathogenic or not. Among 438 strains from birds, 57.6% (49 out of 85) group D strains were *aatA*-positive, while a lower percentage was calculated for groups A (29.7%; 41/138), B1 (39.5%; 30/76), and B2 (34.3%; 48/140).

To get an idea about the distribution of the three different *aatA *"variants" as determined by the variable flanking regions observed by sequencing and *in silico *analyses, a set of 148 *aatA*-positive strains were analysed by three PCRs using primers specifically binding to the internal part of *aatA *and to the respective flanking genes *fecI *(IMT5155; variant 1), *ykgN *(BL21; variant 2), and *eitD *(APEC_O1; variant 3), the latter one likely reflecting a plasmid localization of *aatA *in the respective positive strains. Most of the strains tested harboured *aatA*-flanking variant 1 (21.6%) and variant 2 (18.2%), both putatively resembling a chromosomal location of *aatA *in these strains. On the contrary, the APEC_O1 episomal variant 3 was only observed in 6.8% of the strains. More than 50% of the strains were negative for all three variants tested, indicating the presence of yet other regions flanking the *aatA *gene, which remain to be determined.

## Discussion

The pathogenesis of *E. coli *is a multifactorial process depending on a variety of pathogenicity factors. A vast amount of already known and still unknown virulence determinants defines the virulence of a certain strain and thus the strength of the disease symptoms induced in the corresponding host organism. Although recent studies revealed considerable intersection between ExPEC pathotypes in general, the set of virulence genes present in pathogenic strains can differ considerably in terms of number and combination of genes [7,8,21]. Thus, the identification and characterization of additional virulence associated factors would still improve our understanding of the mechanisms underlying the pathogenicity and virulence of a certain group of *E. coli *strains.

Making use of two clinical strains, namely IMT5155 and CFT073, which differ with respect to host (avian versus human), pathotype (APEC vs. UPEC), O-type (O2 vs. O6), and multilocus sequence type (STC95 vs. STC73) in an SSH approach we identified an *E. coli *adhesin of the autotransporter family. The method of SSH enabled us to determine genes of the so far not sequenced APEC strain IMT5155 representing a well studied prototype strain isolated from a chicken in a German poultry flock which had experienced a severe outbreak of systemic *E. coli *infection [10,16]. At the beginning of our studies, no sequence information was available for any APEC strain. Thus, SSH promised to be a useful tool to achieve sequence information about specific genes present in the avian pathogen but not in the human UTI strain albeit both being ExPEC strains. Indeed SSH has successfully been used in the past in many aspects, including the identification of virulence genes [22-25].

Among 28 DNA fragments that were specific for IMT5155 in our SSH approach, a 225 bp fragment, which showed similarity to putative adhesins, attracted our attention. Although in the run of our experiments a 98% identical adhesin gene as well as the functional role of its product *in vitro *and *in vivo *have been published by Li and colleagues [17], we still considered it important to complete our data as we observed some essential differences to the mentioned study.

Adhesins are involved in the first step of infection, allowing the primary and intimate contact of the pathogen with its host cell, initiating a pathogenic cascade. Their localization at the bacterial surface and their supposed function for enhanced gut colonization in terms of an intestinal reservoir make adhesins attractive candidates for vaccine developmental strategies [16].

APEC strain IMT5155 harbours the *fim *genes for the type 1 fimbriae, *csg *genes for curli fibers and the temperature-sensitive haemagglutinin (*tsh*) gene [16]. It is interesting that IMT5155 lacks P, F1C, S and Dr fimbriae, known to be specifically involved in UPEC pathogenesis [16,26,27]. Thus, other, so far unidentified adhesins might play a role in IMT5155 pathogenesis. Indeed, we recently identified the *yqi *gene cluster encoding a fimbrial type of adhesin, called EA/I, that has been shown to confer an adhesive phenotype to a *fim *negative K-12 strain [16]. Our data presented here show that autotransporter adhesin AatA might also play a certain role in the pathogenesis of APEC infections. In fact, few autotransporter type adhesins have been shown to be involved in APEC virulence to date. In 1994, Tsh which confers agglutination of chicken erythrocytes, was identified in APEC strain χ7122 [28]. Later, Dozois and co-workers showed that Tsh probably contributes to the development of air sac lesions in birds [19]. Furthermore, it turned out that the vacuolating autotransporter toxin Vat, identified in APEC strain Ec222 for the first time, was involved in the development of cellulitis in broiler chickens [29]. Comparable to Tsh and Vat, AatA of APEC IMT5155 comprises all structural motifs characteristic for members of the family of autotransporter proteins: a signal peptide at the N-terminus, which would be recognized by the Sec secretion machinery; an autotransporter repeat, a passenger domain and a C-terminal translocation domain were predicted.

Adherence inhibition assay with a fusion protein containing the central part of the AatA protein confirmed the adhesive properties of AatA. This central part comprises the passenger domain, which is the secreted and surface-exposed protein part and thus the protein domain with supposed virulence function. While the translocation domain is highly conserved, the passenger domain demonstrates considerable sequence variation [12] making it a good candidate to gain specific antibodies against AatA.

By quantitative real-time PCR and immunoblot assays we could show that IMT5155 and APEC_O1 wildtype *aatA *are expressed under lab conditions, which stands in contrast to what Li *et al. *(2010) stated for APEC_O1 in their recent publication [17]. These contradictory observations might in part be explained by different procedures used for antibody production, which could have led to a better detection of the AatA wild-type protein in our study. The failure to detect the very similar BL21-AatA protein with our antibody could be due to the low transcript level as indicated by qPCR experiments. Lower transcription might in turn have occurred due to sequence changes in the promoter regions in front of the IMT5155 and BL21 *aatA *ORFs, which in fact show only 70% identity. Adhesion assays with the *fim *negative strain AAEC189 expressing the IMT5155 AatA confirmed not only the adhesive properties for AatA but also the functionality of the predicted native promoter region. In addition, adhesion inhibition assays indicated a role for AatA as adhesin for IMT5155, which substantiates the findings of Li *et al. *[17] and indicates that the location of *aatA*, either on a plasmid or on the chromosome, does not seem to have any influence on the function of the adhesin, which has to be further investigated in the future.

The ability of bacteria to adhere to a diverse range of surfaces including different host tissues and abiotic elements is essential for colonization, survival and persistence [30,31]. This is demonstrated by the enormous number of different adhesins known so far. It is assumed that a bacterial cell has such a huge set of diverse adhesive proteins to be able to adhere to different tissues and surfaces [15,31]. Indeed the results of our adhesion inhibition assays supported this idea as blocking of IMT5155 and of DF-1 cells did not have a relevant effect on the adhesion property, showing that other adhesins are still effectively mediating adhesion.

An involvement of AatA in adhesion does not necessarily predict its vital importance for the virulence of a strain *in vivo*. However virulence, in particular with regard to ExPEC strains, is often a result of the interplay of several factors, with adhesion-related factors representing one of the most essential groups. Here, a number of adhesins are involved making it difficult to assess the contribution of one single adhesin to disease symptoms. However, for the 98% identical AatA of APEC_O1 its contribution to full virulence in chicken was shown [17].

One simple view is that one adhesin specifically mediates the adhesion to one specific receptor on the eukaryotic cell. This assumption led to the question if AatA isolated from APEC IMT5155, which enters the chicken via the respiratory tract, specifically recognizes proteins of the avian trachea and lung tissue. Interestingly, deduced from the amino acid sequence, AatA clustered together with Pertactin from *B. pertussis*, an adhesin which mediates binding to the lung epithelium of mammals (Figure 3; [32,33]). As this is just a presumptive sequence-based finding, the identification of the host tissue receptor and its interaction with AatA has to be explored in future studies.

A number of publications claim that autotransporter adhesins are of special interest as they constitute an essential component of vaccines used in the medical area [12]. Pertactin from *Bordetella pertussis *was the first autotransporter adhesin used as a vaccine [34]. Also for Hap from *H. influenzae *elicitation of specific antibody titres was shown in mice [35]. Indeed, new classes of these drugs are needed because of the increasing incidence of pathogenic organisms resistant to conventional antimicrobial agents; and it is believed that strains with genotypic resistance to the anti-adhesion agents will spread much slower than strains resistant to conventional drugs, such as antimicrobial substances [36]. Here, we show that specific antibodies can be produced against AatA. Furthermore, we performed prevalence studies to verify if AatA fulfils criteria to serve as vaccine component from an epidemiological point of view. In contrast to the previously described novel adhesin gene *yqi*, initially identified in APEC strain IMT5155 [16], *aatA *was significantly associated with avian isolates, in that more than 90% of all positively tested strains were APEC and avian commensal strains, respectively, which is in accordance with the findings of Li *et al. *[17]. Envisioning an intestinal prevention strategy that aims to combat pathogenic strains from colonizing the proposed intestinal reservoir, the frequent presence of *aatA *in avian commensal strains would basically contradict this idea, as the biological function of the physiological microbiota, including that of non-pathogenic *E. coli *strains, should not be diminished by such a vaccine. However, a high percentage of *aatA *positive strains was allocated to phylogenetic groups B2 and D. Avian commensal strains belonging to these groups have recently been shown to harbour an essential set of virulence genes and to be pathogenic for chickens [37]. Thus, they represent pathogenic strains residing in the chicken intestine rather than fulfilling the criteria of non-pathogenic strains. In conclusion, AatA might not only be relevant to the adhesion of the upper respiratory tract of birds and subsequent pathogenic processes but seems to promote intestinal colonization, thereby contributing to the maintenance and transmission of pathogenic strains. A similar situation could be imagined for *aatA *positive *E. coli *strain B_REL606 that has been isolated from the human gut, but to our knowledge has not undergone further characterization in terms of potential extraintestinal virulence so far.

Li and colleagues found a significant association of *aatA *with isolates assigned to phylogenetic group D with 70% of APEC strains from this phylogenetic group being *aatA*-positive, and more than half of all *aatA*-positive strains belonging to phylogenetic group D [17]. We observed a similar situation among our strain collection, while a distinction between different *aatA*-flanking region variants revealed that variant 1 (IMT5155) was more frequently observed in group B2 and D strains and variant 2 (BL21/B_REL606) in group A and D strains, while, although only rarely detected, the presumed episomal *aatA *variant 3 (APEC_O1) was linked with group B2 strains. Further large-scale analysis will have to rule out, whether the distribution of different *aatA*-flanking variants may be influenced by the phylogenetic background of the strains or by selective forces driven by environmental conditions, e.g. given in a certain host compartment.

The overall wide distribution of this supposed virulence associated gene in a large range of ExPEC and commensal isolates is indicative for a horizontal transfer of *aatA*. It is well established that virulence factors are often located on mobile elements, such as plasmids or pathogenicity islands and are thus often subjected to horizontal gene transfer [4]. Sequence analyses of *aatA *and the flanking regions revealed a potential of mobility for the adhesin gene. In all completely sequenced *E. coli *genomes, where an *aatA *sequence was detected, the gene locus was enclosed by transposable elements. Furthermore, episomally located *aatA *variants might be transferred in the context of the whole plasmid, presuming the presence of functional transfer and mobility elements.

In addition, possible sequence variations among *aatA *genes of strains allocated to different phylogenetic groups might be reflected functionally, which has for example been shown for the genes of the *fim *cluster [38]. Since *aatA *was retained in isolates of different phylogenetic groups, the discrete function of the protein in the respective strains, whether they commensally colonize the intestine or invade other internal organs of poultry and cause severe systemic infections, remains unsolved to date and should be subjected to thorough investigations in the future.

Many autotransporter adhesins are known to be relevant not only for adhesion but also for biofilm formation, invasion, aggregation and toxicity [13]. Adhesins related to AatA, such as Hap, Ag43, AIDA and TibA, for example, contribute to bacterial aggregation by intercellular passenger domain interactions [39]. Most trimeric autotransporter adhesins also seem to confer serum resistance by binding to components of the complement system [40]. Although IMT5155 does not produce a biofilm under normal lab conditions, it remains to be determined if *in vivo *conditions might probably trigger this phenotype, enabling to investigate a possible role of AatA in this process. Although Li *et al. *suggested that AatA is not involved in autoaggregation or biofilm formation [17], it did not become evident whether they tested the wild-type and mutant strain, observing no difference, or whether the wild-type strain APEC_O1, comparable to IMT5155, did not show these phenotypes in general.

## Conclusion

A chromosomal variant of the autotransporter adhesin gene *aatA*, which has recently been described in the plasmid pAPEC-O1-ColBM of APEC_O1 [17] was identified in APEC strain IMT5155. The gene product conferred adhesion of a *fim*-negative K-12 strain to DF-1 cells and its passenger domain was able to trigger immune responses in rabbits. Prevalence studies clearly hinted towards a special importance of this adhesin in avian pathogenic *E. coli *strains, whether outbreak or so-called reservoir strains, while an essential functional role for other animal and human ExPEC strains cannot be inferred from the present data. Different flanking regions as well as mobility elements indicate a high frequency of horizontal gene transfer of *aatA*, the driving forces which have yet to be determined.

## Methods

### Bacterial strains and growth conditions

For Suppression Subtractive Hybridization (SSH) we used APEC strain IMT5155 (O2:K1:H5) [10] and human UPEC strain CFT073 (O6:K2:H5) [41]. IMT5155 was isolated in 2000 from the internal organs of a laying hen in Germany with clinical symptoms of septicemia. It has been included in large-scale phylogenetic analysis and was grouped into one of the most dominant lineages, namely phylogenetic group B2 and multi locus sequence type (ST) 140 of ST complex 95 complex [10,37,42]. Chicken infection studies using a systemic infection model [43] showed that APEC strain IMT5155 as well as UPEC strain CFT073 cause severe symptoms of systemic infection in 5-week-old SPF chickens and can be isolated from all internal organs in comparable numbers (C. Ewers, unpublished data). Non-pathogenic *E. coli *K-12 strain was used as control strain in SSH analysis.

To determine the distribution of the putative adhesin gene *aatA *among ExPEC and commensal *E. coli *strains, a strain collection (n = 779) available at the Institute of Microbiology and Epizootics, Freie Universität Berlin (n = 691), and at the College of Veterinary Medicine, Nanjing Agricultural University (n = 88) was screened. The strain set included 336 APEC, 149 UPEC, 25 newborn meningitis-causing *E. coli *(NMEC), and 44 pathogenic strains from diverse extraintestinal locations, referred to as "other ExPEC". The majority of ExPEC strains originated from birds (n = 336), companion animals (n = 90), and humans (n = 89). In addition, a total of 225 commensal strains from humans (n = 89), birds (n = 103), and from non-avian animal sources (n = 33) were included.

*E. coli *DH5α was used for cloning procedures, BL21(DE3)pLysS was included in protein expression analysis [44] and the *fim *negative *E. coli *strain AAEC189 [20] was used for adhesion assay experiments. All *E. coli *strains were grown at 37°C in LB medium, supplemented with ampicillin (100 μg/ml LB), where necessary.

### Suppression Subtractive Hybridization (SSH)

SSH was carried out between APEC strain IMT5155 and UPEC strain CFT073 using Clontech PCR-Select™ Bacterial Genome Subtraction Kit (Clontech, Heidelberg, Germany) according to the manufacturer's manual. Briefly, genomic DNA (1.5-2.0 μg/subtraction) of IMT5155 and CFT073 served as tester and driver DNA, respectively. The extracted genomic DNA of tester and driver was digested with restriction enzyme *RsaI*. Tester DNA was subdivided into two portions, which were then ligated with Adaptor 1 and Adaptor 2R, respectively, provided with the kit. After that, two hybridizations were performed. First, an excess of driver DNA was added to each adaptor-ligated tester sample. The samples were then heat-denatured and allowed to anneal. During the second hybridization, the two primary hybridization samples were mixed together without denaturing. Thus, the new hybrids were double-strand tester molecules with different ends. Freshly denatured driver DNA was added to further enrich the tester-specific sequences. The entire population of molecules was then subjected to PCR to amplify the desired tester-specific sequences using the primer corresponding to the T7 promoter sequence located in the adaptors. Only tester-specific sequences with two different adaptors are amplified exponentially. A second PCR amplification was performed using nested primers to further reduce any background PCR products and enrich for tester-specific sequences. The resulting PCR products which were assumed to represent tester-specific DNA were cloned into plasmid pCR2.1 using the TOPO-TA cloning kit (Invitrogen, Germany) according to the manufacturer's recommendations.

### Southern blot

Southern blot was performed using Roche^® ^DIG DNA Labelling and Detection Kit (Roche, Shanghai, China) to prove whether the DNA fragments cloned into plasmid pCR2.1 were present in the genome of CFT073 and MG1655 or not. First, the genomic DNA of the strains CFT073 and MG1655 was labelled by random primed labelling with digoxigenin according to the manufacturers manual. PCR products of the subtractive clones were transferred onto two identical positively charged nylon membranes. Hybridizations were performed using the labelled genomic DNA of the strains CFT073 and MG1655, respectively. Chemiluminescent substrate reactions were carried out using the antidigoxigenin-AP Fab fragments and visualized with the CSPD ready to use (Roche, Shanghai, China).

### Cosmid library

The cosmid library from APEC strain IMT5155 was created using the SuperCos 1 Cosmid Vector Kit (Stratagene, Amsterdam, Netherlands) following the vendor's recommendations.

### DNA extraction

Genomic DNA and cosmid DNA was isolated using standard protocols [45]. Plasmid DNA was isolated using the High Pure Plasmid Isolation Kit (Roche, Mannheim, Germany). PCR products were purified using the High Pure PCR Product Purification Kit, and DNA extraction from agarose gels was performed using the Agarose Gel DNA Extraction Kit (Roche, Mannheim, Germany) according to the manufacturer's guidelines.

### PCR detection of aatA and flanking region variants in E. coli

The screening for *aatA *in a collection of 779 *E. coli *strains was performed by standard PCRs targeting three regions of the entire gene (amplicons A, B, and C). Oligonucleotide sequences (4031 to 4036) are listed in Additional file 1: Table S1, whereas their localization within the *aatA *ORF and respective amplicon sizes are given in Figure 1A. IMT5155 was used as a positive control, while CFT073 served as a negative control for all PCRs. To determine the genomic localization variants of *aatA *homologs in different strains, oligonucleotides aatA-FP and fecI-RP, eitD-RP and ykgN-RP were used in PCR experiments, respectively (Additional file 1: Table S1).

Genomic DNA was used as template and 0.5 μl were added to a 25 μl reaction mixture containing the following: 0.5 μl of each primer pair in a 10 pmol concentration, 0.5 μl of a 10 mM desoxynucleoside triphosphate mixture (Sigma-Aldrich Chemie GmbH, Munich, Germany), 2.5 μl of 10x PCR buffer, 2 μl of 50 mM magnesium chloride and 1 unit of *Taq*-Polymerase (Rapidozym GmbH, Berlin, Germany). The samples were subjected to 25 cycles of amplification in a thermal cycler (GeneAmp PCR system, Applied Biosystems, Darmstadt, Germany) with an annealing temperature predicted by the respective oligonucleotides calculating an extension time of 1 min per 1 kb. Amplification products were analysed by gel electrophoresis on a 1% agarose gel (Biodeal, Markkleeberg, Germany), stained with ethidium bromide and photographed on exposure to UV.

### ECOR typing of the strain collection

Subgroups of single isolates were determined by a triplex-PCR as described previously [46].

### DNA sequence analysis

Sequencing of PCR fragments and cosmid clones was performed on an ABI PRISM 377 XL DNA Sequencer (Perkin-Elmer, Massachusetts, USA). Sequences were analysed using online programs (BLASTN and BLASTX) in GenBank http://www.ncbi.nlm.nih.gov/blast/. To sequence *aatA*, the cosmid region of the IMT5155 library containing *aatA *was commercially sequenced (LGC Genomics, Berlin, Germany) and obtained sequences were analysed using the alignment tool of the BioNumerics software (V.4.601; Applied Maths, Belgium). Promoter prediction analyses were carried out with prediction program tools, available at http://www.cbs.dtu.dk/services/Promoter/.

### Protein sequence analysis

For phylogenetic analyses of autotransporter proteins, ClustalW analyses were performed using http://align.genome.jp/. Protein sequences were obtained from the NCBI database http://www.ncbi.nlm.nih.gov/protein. Pylogenetic N-J trees were obtained using complete or partial protein sequences, respectively.

### Expression and purification of the putative adhesin AatA

Using oligonucleotides B11-for and B11-rev (Additional file 1: Table S1; Figure 1), the central fragment (1,222 bp) of the putative adhesin gene was amplified by PCR adding *BamHI *and *XhoI *recognition sites. The obtained PCR fragment was digested with these two enzymes followed by ligation into *BamHI*/*XhoI*-digested pET32a(+) vector (Novagen, Shanghai, China). The resulting plasmid pET32a:*aatA*_1222, which allows the expression of a fusion protein controlled by an IPTG-inducible promoter was transformed into competent *E. coli *BL21(DE3)pLysS cells by heat shock transformation. The expression of AatAF was induced by adding IPTG with a final concentration of 1 mM to the culture. Protein purification was performed using a HisTrap HP column (GE Healthcare, Shanghai, China) according to the manufacturer's guidelines. Purified AatAF protein was dialyzed overnight at 4°C against 500 ml of dialysis buffer (50 mM sodium phosphate, pH 7.5) followed by a concentration step using Amicon Ultra-4 filter (10 000-Da cutoff; Millipore). The final protein concentration was determined by the Bradford method using SmartSpec3000 (Bio-Rad, Shanghai, China) [47]. Expression and purity of the fusion protein was determined by SDS-PAGE according to standard protocols [45]. Immunoblot analysis was performed as described by Ausubel *et al. *(1996) using anti-AatA antibody (see below).

### Antibody production

The anti-AatA antibody was produced in New Zealand White rabbits as follows: 300 μg highly purified fusion protein solved in PBS were mixed with an equal volume of adjuvant ISA 206 (SEPPIC S.A., Puteaux, France) and subcutaneously injected into the back of the rabbits at seven different sites. Immunization was repeated thrice at 2-week intervals. Ten days after the final immunization blood was collected by cardiac puncture under terminal anaesthesia, and serum samples were prepared and frozen at -20°C.

### Quantitative real-time PCR

Overnight cultures of *E. coli *were diluted to an OD_600 _= 0.1 in fresh LB. Bacteria were grown to the logarithmic phase (OD_600 _= 0.8), harvested, and cell pellets were resuspended in Trizol (Invitrogen GmbH, Karlsruhe, Germany). Total RNA was isolated according to the manufacturer's protocol followed by digestion of the genomic DNA using RQ1 RNase-Free DNase (Promega, Mannheim, Germany). cDNA synthesis was then performed using random hexamere-primers and the MMLV reverse transcriptase following the manufacturer's protocol. cDNA aliquots corresponding to 150 ng of total RNA were semi-quantitatively analyzed using sense (aatA RT-F) and antisense oligonucleotides (aatA RT-R) of the target gene *aatA *and analyzed by real-time PCR (Applied Biosystems StepOne) with the SYBR^® ^Green method. The relative gene expression of *aatA *was normalized to the expression of the housekeeping gene *gyrB*, which was amplified using primers 4057 and 2521 (Additional file 1: Table S1), via the ΔΔCt method. PCR efficiency (> 90%) for each of the gene was checked via standard dilution curves.

### Immunoblot

For immunoblot experiments, overnight cultures of *E. coli *were diluted 1:100 into fresh LB. The bacteria were grown to the logarithmic phase, harvested, resuspended in protein denaturation buffer and boiled for 10 min [48]. Total protein extracts were loaded on 10% SDS gels and transferred onto a polyvinylidene fluoride membrane (Amersham Pharmacia Biotech, Shanghai, China) using a semi-dry blotting apparatus (TE77, Amersham Pharmacia Biotech) and a buffer containing 39 mM glycine, 48 mM Tris base, 20% methanol, and 0.037% SDS. Serum raised against the passenger domain of AatA was used as primary antibody and horseradish peroxidase-conjugated antirabbit immunoglobulin as secondary antibody. Tetra methyl benzidine was used as the substrate to visualize protein bands.

### Adherence assay

For adhesion studies, the IMT5155 *aatA *ORF and the 99 bp upstream containing the putative native *aatA *promoter were amplified and cloned into pMD18T (TaKaRa, Dalian, China) vector using oligonucleotides WSH18F and WSH16R adding the restriction enzyme recognition sites *BamHI *and *HindIII*. The obtained construct pMD18T:*aatA*+P was transformed into *E. coli *DH5α by electroporation. Plasmids pMD18T:*aatA*+P and pUC18 were digested with restriction enzymes *BamHI *and *HindIII *and the *aatA+P *fragment was ligated into pUC18. The empty vector pUC18 and plasmid construct pUC18:*aatA *were transformed into AAEC189 resulting in AAEC189(pUC18) and AAEC189(pUC18:*aatA*_+P_), respectively.

Chicken embryo fibroblast DF-1 cells were seeded with about 1 × 10^5 ^cells per well in 24 well tissue culture trays (TPP, Shanghai, China). Cells were grown in DMEM with 10% fetal bovine serum (Invitrogen, Shanghai, China) at 37°C in a 5% CO_2 _humidified atmosphere and incubated for 36 h prior to adherence assays. Semiconfluent monolayers were washed and incubated with DMEM without fetal bovine serum. *E. coli *strains used for infection of the DF-1 cells were grown to logarithmic phase and harvested by centrifugation. After washing in PBS (pH 7.4), bacteria were resuspended in DMEM without fetal bovine serum. Bacteria were then inoculated into wells containing monolayers of DF-1 cells to a final MOI of 100. Infected monolayers were incubated for 3 h at 37°C under 5% CO_2 _atmosphere to allow the bacteria to adhere to the cells. After 3 h incubation, the DF-1 cell layers were washed three times with PBS. Cells were incubated with 1% Triton X-100 and bacterial cells were diluted in PBS and plated on LB agar plates in dilution series. After incubation at 37°C over night numbers of colonies were determined. Results were expressed as the average number of bacteria adhering to DF-1 cells. Negative control wells containing only DF-1 cells were used in all experiments.

For adherence inhibition experiments, the purified protein AatAF was refolded and 50 μg were added to each well containing a DF-1 cell monolayer. As a control experiment DF-1 cells were incubated with 50 μg bovine serum albumin (BSA) per well. After 1 h incubation at 37°C, DF-1 cell monolayers were washed with PBS and bacterial cells of strain IMT5155 were added with an MOI of 100. Adherence assays were done as described above.

To analyse the effect of anti-AatA to the adherence of IMT5155, bacteria were pre-treated with specific anti-AatA antibody and pre-immume serum for 1 h at 37°C. Pre-treated bacteria were used to infect DF-1 monolayers as described above. The assay was performed three times in duplicates.

### Statistical analysis

Statistical analysis for *in vitro *cell culture experiments were carried out using the software SPSS (Version 17.0; SPSS Inc., IL, USA) by carrying out the non-parametric Mann-Whitney U-Test and the students *t*-test at the 95% significance level (*p *< 0.05).

Significance of associations between *aatA *and pathotypes, host and ECOR groups, respectively, was determined by applying a χ2 test, using PASW Statistics 18 (SPSS Inc., Chicago, IL, USA). *P*-values *p* < 0.001 were considered significant.

## Authors' contributions

JD and CL: carried out basic SSH screening, SW carried out sequencing, antibody production, adhesion and adhesion inhibition assay and PCR screening for prevalence studies, DG did sequencing analyses, *in silico *analyses, supervised laboratory work of SW and created figures and the final version of the manuscript, SG performed real-time PCR analyses, ZS contributed to adhesion assays, CPL supervised JD and SW and was responsible for a first draft of a manuscript, CE performed experimental and statistical analyses of the distribution of *aatA *and its flanking region, supervised the work of SW, and strongly contributed to the final version of the manuscript. All authors read and approved the final manuscript.

## Supplementary Material

Additional file 1 **Oligonucleotide primers used in this study**. Names and nucleotide sequences of oligonucleotide primers used in this study. Click here for file
