# Supplementary material for: Suppression subtractive hybridization identifies an autotransporter adhesin gene of E. coli IMT5155 specifically associated with avian pathogenic Escherichia coli (APEC)
Source: BMC Microbiol. 2010 Sep 9;10:236. doi: 10.1186/1471-2180-10-236 (PMC2944236; doi:10.1186/1471-2180-10-236)
Supplement: Additional file 1 — Oligonucleotide primers used in this study. Names and nucleotide sequences of oligonucleotide primers used in this study. [file 1471-2180-10-236-S1.DOC]

**Table S1:** **Oligonucleotides used in this study**

| **Oligonucleotide primer** | **5’→ 3’ sequence** |
| --- | --- |
| B11up | GCCGAGGTACTCTTATACTTTCT |
| B11down | CCGTCAGTAGTTTGCTTATTGTC |
| 4031 | atgaataagaatatacgaattttac |
| 4032 | accattattatttagcgtaaag |
| 4033 | cataggcgtttctctttccgat |
| 4034 | cctgtcgttcatacagattcgtt |
| 4035 | ctgctgagtcatggaagtcaa |
| 4036 | tcagaaacgatattccacgctc |
| 4035 | ctgctgagtcatggaagtcaa |
| 3885 | GAAGACGGTTGTTGCGCAC |
| B11-for | AGCGGATCCATAGGCGTTTCTCTTTCC |
| B11-rev | ATGCTCGAGGTCGTTCATACAGATTCG |
| 3808 | ATGAATAAGAATATACGAATTTTAC |
| 3809 | ACCATTATTATTTAGCGTAAAG |
| 3810 | CATAGGCGTTTCTCTTTCCGAT |
| 3811 | CCTGTCGTTCATACAGATTCGTT |
| 3812 | CTGCTGAGTCATGGAAGTCAA |
| 3813 | TCAGAAACGATATTCCACGCTC |
| 3883 | caacctgaagctgtaccg |
| 3884 | cattcatccggacaacctg |
| 3886 | CTTCCATTTCCATCAGATGTC |
| 4039 | gagcaggtgcactgaacattag |
| 4040 | Cagggagtatcacgagacg |
| WSH 18F | GCAGGATCCTTTCTATTGAGAAAAAACA |
| WSH 16R | CGCAAGCTTCAGAAACGATATTCCAC |
| 4057 | ctgcggtacaccgtgttc |
| 2521 | TCGGCGACACGGATGACGGC |
| aatA RT-F | CCGTACCCGTGTCGCTGTTAC |
| aatA RT-R | CAGCATTATCAGCATTGCCACT |
| aatA-FP | TGATGCAGGCATTCAACTTGG |
| fecI-RP | CGTTAAGGAAGCTGTGGTAGT |
| eitD-RP | GTTTATCCCTGGCTTCTGCT |
| ykgN-RP | GGGATAACGCTTACCGCTCA |
